# Graphene Nanoribbons: Prospects of Application in Biomedicine and Toxicity

**DOI:** 10.3390/nano11092425

**Published:** 2021-09-17

**Authors:** Olga V. Zakharova, Elena E. Mastalygina, Kirill S. Golokhvast, Alexander A. Gusev

**Affiliations:** 1 Research Institute for Environmental Science and Biotechnology, Derzhavin Tambov State University, 33 Internatsionalnaya St., 392000 Tambov, Russia; olgazakharova1@mail.ru; 2 Engineering Center, Plekhanov Russian University of Economics, Stremyanny Lane 36, 117997 Moscow, Russia; elena.mastalygina@gmail.com; 3 Department of Functional Nanosystems and High-Temperature Materials, National University of Science and Technology MISiS, 4 Leninskiy prospekt, 119049 Moscow, Russia; 4 Laboratory of Physics-Chemistry of Synthetic and Natural Polymers Composites, Institute of Biochemical Physics Named after N.M. Emanuel RAS (IBCP RAS), Russian Academy of Sciences, 4 Kosygin St., 119991 Moscow, Russia; 5 Polytechnical Institute, Far Eastern Federal University, Sukhanova 8, 690950 Vladivostok, Russia; droopy@mail.ru; 6 Siberian Federal Scientific Center for Agrobiotechnology RAS, Centralnaya 2B, 630501 Krasnoobsk, Russia; 7 Pacific Geographical Institute, Far Eastern Branch of the Russian Academy of Sciences, Radio 7, 690041 Vladivostok, Russia; 8 Research Educational Center Sustainable Development of the Forest Complex, Voronezh State Forestry University Named after G F Morozov, 394087 Voronezh, Russia

**Keywords:** graphene nanoribbons, biomedical application, sensors, gene delivery, drug delivery vehicles, graphene nanoribbons toxicity

## Abstract

Graphene nanoribbons are a type of graphene characterized by remarkable electrical and mechanical properties. This review considers the prospects for the application of graphene ribbons in biomedicine, taking into account safety aspects. According to the analysis of the recent studies, the topical areas of using graphene nanoribbons include mechanical, chemical, photo- and acoustic sensors, devices for the direct sequencing of biological macromolecules, including DNA, gene and drug delivery vehicles, and tissue engineering. There is evidence of good biocompatibility of graphene nanoribbons with human cell lines, but a number of researchers have revealed toxic effects, including cytotoxicity and genotoxicity. Moreover, the damaging effects of nanoribbons are often higher than those of chemical analogs, for instance, graphene oxide nanoplates. The possible mechanism of toxicity is the ability of graphene nanoribbons to damage the cell membrane mechanically, stimulate reactive oxidative stress (ROS) production, autophagy, and inhibition of proliferation, as well as apoptosis induction, DNA fragmentation, and the formation of chromosomal aberrations. At the same time, the biodegradability of graphene nanoribbons under the environmental factors has been proven. In general, this review allows us to conclude that graphene nanoribbons, as components of high-precision nanodevices and therapeutic agents, have significant potential for biomedical applications; however, additional studies of their safety are needed. Particular emphasis should be placed on the lack of information about the effect of graphene nanoribbons on the organism as a whole obtained from in vivo experiments, as well as about their ecological toxicity, accumulation, migration, and destruction within ecosystems.

## 1. Introduction

Graphene nanoribbons (GNRs) are narrow strips of graphene composed of repeating hexagonal carbon cells, up to 50 nm wide and up to several dozens of micrometers long, depending on the synthesis method [1]. Graphene ribbons were theoretically described in 1996 by Fuhita et al. as a model for studying the edge and nanoscale effects of graphene [2,3]. Due to their quasi-one-dimensional nature, GNRs differ significantly from the more widely known two-dimensional graphene sheets [4]. Being almost ideal nanowires or nanotags, GNRs are an extremely accurate tool, promising for nanoelectronic components, ultrasensitive chemical and mechanical sensors, etc. [5,6,7,8,9,10].

Moreover, the structure and physical properties of GNRs vary significantly, depending on the synthesis method. In fact, GNRs obtained via different methods are very different and have little in common. Today, three main approaches to obtaining GNRs exist [11]:

1. Electron-beam lithography and photolithography: This method makes it possible to obtain single-layer GNRs on the substrate surface; however, the fields of application of lithographically obtained GNRs are very limited. In addition, the ribbons obtained by the lithographic method have jagged edges [12,13,14].

2. Bottom-up synthesis from polycyclic molecules: This method includes multistage organic synthesis based on the cyclization of previously synthesized polymer chains. This method provides very narrow ribbons with an atomically precise edge configuration [15,16,17,18].

3. Unwrapping carbon nanotubes (CNTs): The third approach is based on longitudinal opening or cutting of multiwalled carbon nanotubes (MCNTs) [19,20]. This method produces GNRs with controlled width and well-defined edge structures [21].

Typical characteristics of GNRs are determined by transmission electron microscopy (TEM), atomic force microscopy (AFM), Raman-scattering spectroscopy, and X-ray photoelectron spectroscopy (XPS).

**Figure 1:**
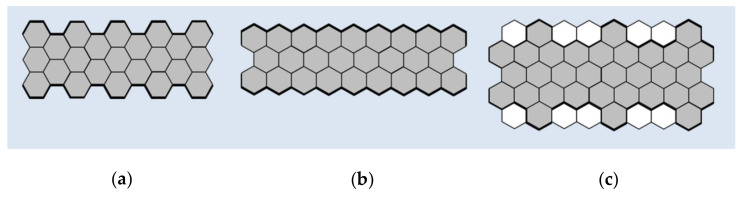
Types of the edge structures of graphene nanoribbons (GNRs): (**a**) “armchair”; (**b**) “zigzag”; (**c**) “cove”.

As previously noted, GNRs can be considered as quasi-one-dimensional graphene strips. This makes them similar to a large class of conjugated polymers, for which the synthesis conditions and the method of film formation determine the performance characteristics [22,23]. The structural perfection of GNRs is a significant problem, because their electronic and optical properties critically depend on the edge configuration, width, and direction of the crystal [24]. The three most commonly studied types of GNRs edge structures are “armchair” or AGNRs, “zigzag” or GNRs with zigzag edges (ZGNRs), and “cove” (Figure 1) [25,26].

GNRs with armchair-type and zigzag-type edges are the most common ones. AGNRs are characterized by a wide band gap that varies with width, while ZGNRs are predicted to have smaller bandgaps with localized edge states that are magnetic and show great potential for spintronic applications [27,28,29,30]. Modifications to the edges of cove-type GNRs are able to smoothly reduce energy bandgaps at the expense of losses in conjugation and increased morphological spreading [31].

Thus, to take full advantage of the exceptional characteristics of GNRs for practical applications, it is necessary to take into account the fabrication method and the type of spatial structure.

## 2. GNRs in Biomedicine

Remarkable electrical and mechanical properties of GNRs make them a promising material for biological and medical applications [32,33,34,35,36]. However, the widespread use of GNRs requires the careful analysis of the potential toxicity of these materials, both through intentional and accidental exposure.

**Figure 2:**
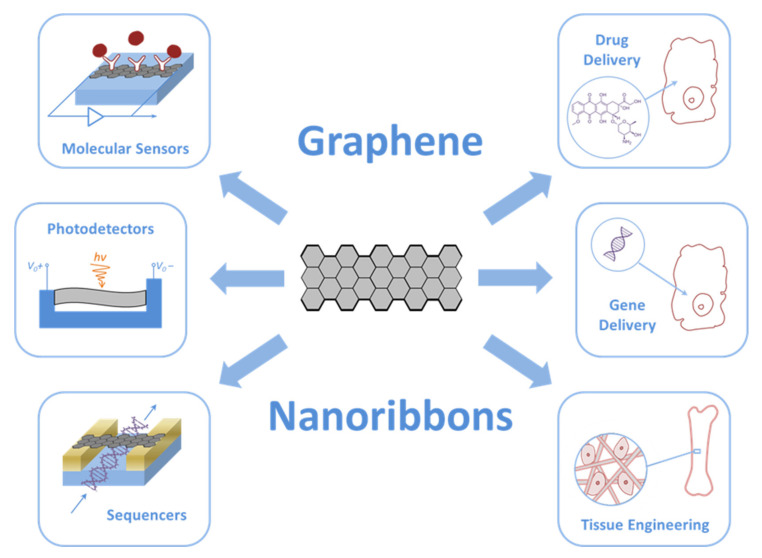
Biomedical applications of GNRs.

According to the available information, GNRs in biomedicine are mainly used to create various ultra-small devices such as molecular sensors, photo-, thermo-, and acoustic detectors, sequencers, drug and gene delivery vehicles, and tissue engineering constructions (Figure 2).

### 2.1. Electronic and Biomedical Devices

The research works aimed at the integration of GNRs into devices are going in several directions, but all of these devices require connections and heterojunctions [22].

Cai et al. successfully manufactured heterojunctions of GNRs with heterostructures by means of combining pristine hydrocarbon precursors with their nitrogen-substituted equivalents. The obtained heterostructures consisted of seamlessly assembled segments of pristine GNRs (p-GNRs) and deterministically nitrogen-doped GNRs (N-GNRs), which behave similarly to traditional p–n junctions. With a band shift of 0.5 eV and an electric field of 2 × 10^8^ V/m at the heterojunction, these materials are applicable for the photovoltaic industry and electronics [37].

The bottom-up synthesis allows obtaining homogeneous GNRs with an extremely narrow width (less than 1 nm) and an atomically perfect edge structure. GNRs of this type are well suited for a wide variety of electronic devices. Bennett et al. developed a reliable layer transfer process for creating nanoscale GNR field-effect transistors by virtue of chemical synthesis. The researchers proved the high sensitivity and good electrical characteristics of the developed GNRs [38].

The studies [39,40,41,42] were aimed at the devices with integrated GNRs, namely photodetectors and sensors. For instance, Johnson et al. obtained hydrogen sensors from Pd-functionalized multilayer networks based on GNRs [42]. The manufacturing method of these networks is cheap and scalable. The developed networks are characterized by a high specific surface area, so they can be used for functionalization and gas adsorption. According to the research results, the networks had high sensitivity to hydrogen at ppm concentration levels at room temperature with fast response and recovery time.

**Figure 3:**
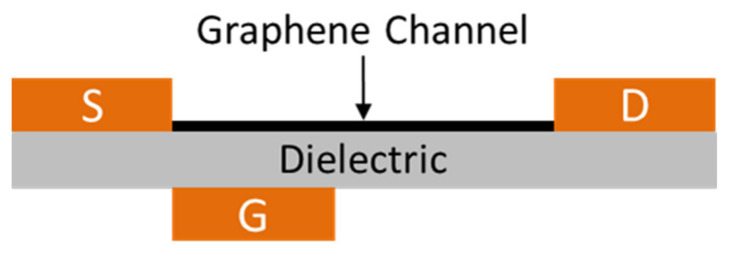
The scheme of the structure of the novel GNR photodetector with one asymmetric metal gate. The width and length of the nanoribbon are 1.35 and 6 nm, respectively. The width of the metal gate is 1/5 of the length of the GNR channel. Adapted from [43].

The basic mechanism in a photodetector is the separation of photo-excited electrons and holes. However, several distinct separation methods have already been discovered, all of which are based on using a longitudinal electric field. Zarei and Sharifi have proposed a novel method based on using a vertical electric field, which induces an asymmetric potential barrier in front of one or both of photo-excited carriers [43]. At the initial stage, the authors used a simple one-dimensional model consisting of a single one-dimensional chain of atoms. The model took into account many aspects, including the influence of the location, height, and width of the potential barrier. For real applications, a new structure based on GNRs and an asymmetric metal gate was developed (Figure 3). The results of the study showed that this structure provides the corresponding separation of carriers.

In the study [44], it has been revealed that the encapsulation of the deposited HfO_2_ on an atomic layer of GNRs significantly increases the mobility of charge carriers and decreases their scattering of their nanoribbons, since the dielectric layer weakens the coulomb interactions of the carriers. In addition, a photodetector based on GNRs coated with a HfO_2_ layer can cover broadband waves from the visible to mid-infrared range at room temperature, demonstrating 10 times higher sensitivity than the one without a HfO_2_ layer in the visible mode and 8 times higher sensitivity in the mid-infrared mode.

DNA detection has received considerable attention in recent years due to promising applications in diagnostics and treatment, forensic science, food safety assessment, etc. Therefore, molecular diagnostic systems for the detection of DNA with high sensitivity and specificity have an enormous potential. For example, solid-state nanopores can act as single-molecule sensors and can potentially be used for quickly sequencing DNA molecules. However, nanopores are usually made in insulating membranes with the thickness up to 15 bases, which makes it difficult to sequence individual bases by such devices. At the same time, graphene has a thickness of only 0.335 nm (equivalent to the distance between two bases in the DNA chain) that provides a suitable membrane for sequencing [45,46,47,48,49].

Nelson et al. first studied the DNA translocations using in-plane current signals in GNRs [50]. The authors analyzed the spectra of conductivity and charge density in the presence of different nucleobases inside graphene nanopores and proved that this device can distinguish four different bases. Meanwhile, the conductivity spectrum of nucleotides is little affected by its orientation inside the nanopores. The proposed technique can be extremely useful for real applications in ultrafast and inexpensive DNA sequencing methods.

**Figure 4:**
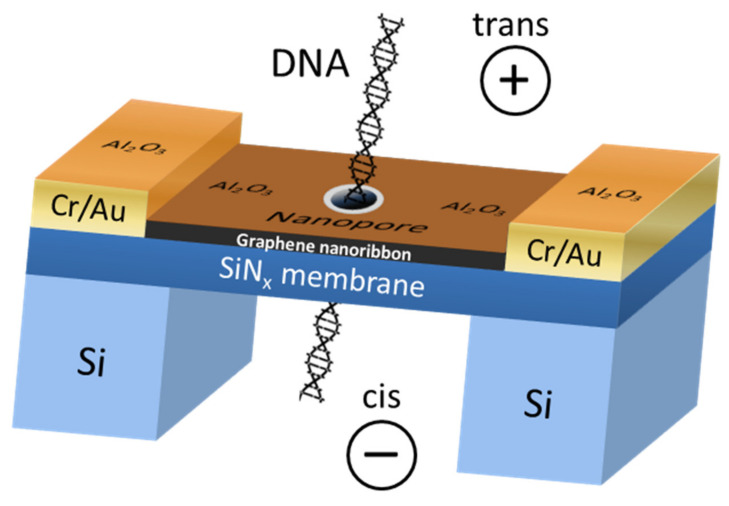
The scheme of the DNA translocation sensor based on GNRs. Adapted from [51].

The first experimental data on DNA translocation through GNR nanopores was reported by Traversi et al. [51]. It was found that a solid-state nanopore could be integrated with a GNR transistor to obtain a sensor for DNA translocation. Figure 4 shows the diagram of the proposed device. As DNA molecules move through the pores, the device can simultaneously measure the ion current drop and local voltage changes in the transistor, which can be used to detect molecules.

When using GNRs based on SiNx membranes, it was shown that the passage of DNA through a nanopore in graphene significantly changes the conductivity of paired nanoribbons. This can be explained by the strong π–π interaction between the atoms of carbon, graphene, and nucleotides in the DNA molecules [52]. Nevertheless, an improved signal-to-noise ratio of graphene electrodes for sequencing single nucleotides is required [53].

Many theoretical studies have predicted that DNA sequencing may be realized by monitoring the transverse current through GNRs while a DNA molecule is translocated through nanopores in ribbons. This type of sequencing benefits from the special transport properties of graphene, provides an ultimate spatial resolution due to the graphene thickness in the monatomic layer and facilitates high-throughput measurements. In the previous experimental attempts to measure such in-plane transverse signals, trivial capacitive response has prevailed. To solve this problem, Heerema et al. [54] used a differential current amplifier, which discriminates between a capacitive current signal and a resistive response in graphene. The authors used short and narrow (30 nm × 30 nm) nanoribbons with 5 nm nanopores obtained using a high-temperature scanning transmission electron microscope to preserve the crystallinity and sensitivity of graphene. It was shown that resistive modulations could be observed in the graphene current due to the DNA translocation through nanopores, thus demonstrating the possibility of measuring DNA using in-plane currents. However, it is worth noting that this approach is challenging due to the low yields in the fabrication of devices associated with a complex multistage device layout.

Puster et al. [55] obtained nanoporous sensors based on graphene nanoribbons (GNR–NP). Pores with a diameter of 2–10 nm were formed at the edge or in the center of a GNR with a width of 20 to 250 nm and a length of 600 nm on a 40 nm silicon nitride substrate. The GNR conductivity was monitored in situ during electron irradiation-induced nanopore formation by a transmission electron microscope operating at 200 kV. It was shown that the GNR resistance increased linearly with increasing electron dose and that conductivity and mobility of GNRs decreased by a factor of 10 or more when GNR images were displayed at a relatively high magnification with a wide beam before creating a nanopore. The TEM analysis in scanning mode (STEM) allows controlling the position of the convergent electron beam with a high spatial accuracy through automatic feedback. Using the STEM mode prevented damage caused by the electron beam that made it possible to create nanopores in the highly conductive GNR sensors. The method used minimizes the effect of GNRs on the beam before and during the formation of nanopores. The resulting GNRs with constant resistances after nanopore formation can withstand microampere currents at low voltages (~50 mV) in a buffered electrolyte solution and exhibit high sensitivity, with a large relative resistance change with gate voltage changes, similar to the original GNRs without nanopores.

**Figure 5:**
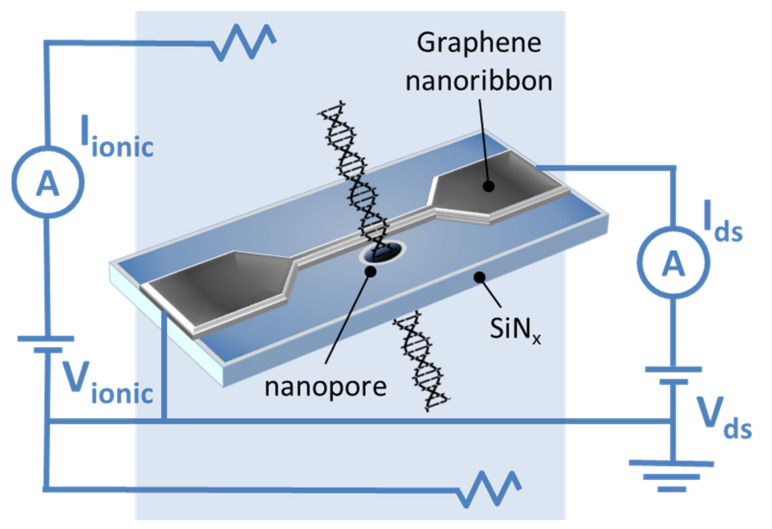
The scheme of the GNR–nanopore structure during DNA translocation. Reproduced with permission from [56]. Copyright John Wiley and Sons, 2015.

In their later work [56], Puster et al. described the scheme (Figure 5) and the technique for measuring ion currents using nanopores, including GNRs up to 50 nm wide and up to 600 nm long, providing a stable ion current linearly dependent on voltage in the open state.

Puster et al. reported that DNA translocation through nanopores occurs when currents from several hundred of nanoamperes to 2 μA at low drain-source voltage (Vds) values (<100 mV) are applied. The DNA translocation modulates both the ionic current through the nanopores and the electronic conductivity of the nanoribbon. Since the ionic signal during DNA translocation usually is a rectangular pulse, the GNR signal can be represented as up- and down-current bursts that occur at the beginning and end of the ion signal. This time-derivative signal is the result of the capacitive coupling between the measurement channels. Crosstalk is not scaled by Vds on the device and is present for the measurements at both high salt (1 M KCl) and low salt (10 mM KCl) concentrations [56].

Saha et al. [57] studied a two-component device for DNA sequencing. The investigated device consists of a metallic ZGNR and a nanopore in its inner part, through which a DNA molecule moves. Using the non-equilibrium Green function method in combination with the density functional theory, it was shown that each of the four DNA nucleic bases inserted into nanopores, in which the edge carbon atoms are passivated by hydrogen or nitrogen, leads to a unique change in the device conductivity. The other recent biosensors based on transverse electron transport through translocated DNA use a small (within the order of picoampere) tunneling current through a nanostructure or nanopore, giving a low signal-to-noise ratio. Contrastingly, the presented device concept is based on the fact that the local-current-density ZGNR reaches a peak at the edges, so drilling the nanopores from the edges will not result in a decrease in conductivity. The insertion of a nucleobase into the nanopore affects the charge density in the surrounding area, thereby modulating the edge conduction currents, the magnitude of which is approximately a microampere at a bias voltage of 0.1 V.

GNRs by functionalized nucleotides are considered as a tool for DNA sequencing in aqueous suspensions. Paulechka et al. [58] proposed a method based on the formation of hydrogen bonds between the nitrogenous bases of the probe and the analyte according to the principle of complementarity. In this case, the selective pairing of nucleotides and the ability of graphene to transform anisotropic deformation of the crystal lattice into changes in the electric current at the nanoscale are combined. Using the methods of modeling atomic and molecular dynamics, the authors estimated the levels of measurable changes in the electrical signal of the nanosensor in response to deformations, taking into account the influence of the environment. From the results, it was concluded that the proposed approach is very promising for the design of DNA sensor devices, allowing obtaining and processing big data with good noise immunity.

GNRs-based electrodes decorated with iron oxide nanoparticles (GNR–Fe_3_O_4_) amplify electrochemical signals by more than one order of magnitude compared to bare carbon electrodes and 70% more compared to p-GNRs-based electrodes [59]. The electrochemical currents in immobilized single-stranded DNA and double-stranded DNA are 92 and 49 μA, respectively. These values are indicative of an effective discrimination between the immobilization and hybridization of DNA.

An electrochemical biosensor on the basis of gold nanocages (AuNCs) and GNRs (AuNCs@GNRs) was developed by Feng et al. [60]. The biosensor is a so-called DNA walker that stochastically moves along the surface, depending on the presence of DNA targets. Due to the large surface area and high conductivity of the biosensor components, its sensitivity is improved. The processes of conjugation and the dissociation of the target DNA with the sensor are more efficient, which leads to a significant amplification of the signal. The proposed biosensor based on AuNCs@GNRs demonstrates excellent characteristics for DNA analysis in complex matrices, such as human serum, which opens up prospects for the practical application of a new sensor platform.

Another example of the use of GNRs in biosensors is described in the study [61]. Mehmeti et al. developed an electrochemical biosensor of glucose based on the composition of enzyme glucose oxidase (GOx) with GNRs, which does not require reagents. Nanoribbons are used for the direct transfer of electrons between the coenzyme, flavine adenine dinucleotide (FAD), and the electrode. The method includes the following stages: purification of GOx by separation from the coenzyme; obtaining GNRs by the oxidative cutting of MCNTs; immobilization of GNRs on the surface of the carbon electrode using screen printing; covalent attachment of FAD to nanoribbons; recombination of the enzyme with covalently linked FAD; stabilization of the bilayer using a thin polymer electrolyte membrane. The resulting biosensor shows operability when using a potential of +0.475 V, and the signals are linearly proportional to the glucose concentration in the range from 50 to 2000 mg/L with a detection limit of 20 mg/L. The biosensor was used to determine glucose in human serum, with a high level of repeatability and reproducibility of the results.

By employing hybrid plasmonic sensing platform with GNR/Ag nanoparticles for the sequential colorimetric detection of dopamine (DA) and glutathione (GSH) in human serum samples was proposed. DA and GSH were successfully detected in low concentrations of 0.04 and 0.23 μM, respectively [62].

Sainz et al. employed chevron-like GNRs to develop a novel electrochemical epinephrine sensor [63]. Pulse voltammetry methods obtain a linear concentration range from 6.4 × 10^−6^ to 1.0 × 10^−4^ M and a detection limit of 2.1 × 10^−6^ M.

Lalwani et al. [64] suggested potential applications of GNRs investigated as contrast agents for photoacoustic and thermoacoustic tomography (TAT). The researchers reported that oxidized single-layer and multiwalled nanoribbons (GO) exhibit a signal amplification for photoacoustic tomography of about 5–10 times compared to lysed bovine blood at a wavelength of 755 nm and ~10–28% signal amplification for thermoacoustic tomography compared to deionized water at a frequency of 3 GHz. Oxidized GNRs are promising in multimodal photoacoustic tomography and TAT contrast agents [65].

Thus, numerous electronic devices based on GNRs have been proposed theoretically and experimentally [46,47,48,66,67,68]. However, the lack of experimental data suggests the need to refine the data on the responses of graphene devices and measure their signals, especially taking into account the previously mentioned differences in the properties of GNRs depending on the methods of fabrication. Table 1 presents potential GNRs applications in biomedical devices.

### 2.2. Delivery of Genes and Drugs

The use of drug delivery systems improves the efficacy of many existing drugs and allows the introduction of new therapies. GNRs are an excellent candidate for drug delivery systems [69] and genes delivery [36].

In the study [70], the analysis of electronic and chemical properties of zigzag nanoribbons functionalized with 1-phenylalanine (C_9_H_11_NO_2_) was conducted using the density functional theory. In particular, properties such as the band gap, charge transfer, chemical potential, dipole moment, bond length and energy, and the characteristic of the s-orbital were investigated. Differences depending on the selection of different functionalization sites were determined. The most chemically active and stable variants in the aquatic environment were identified. This study forms the theoretical basis for the effective use of functionalized nanoribbons as drug delivery vehicles.

Chowdhury et al. [71] studied oxidized GNRs (O-GNRs) as non-viral vectors for gene therapy. It was shown that O-GNRs could load large amounts of small single-stranded or double-stranded genetic materials without additional functionalization with positively charged groups or other non-viral vectors. The used O-GNRs doses of 20–60 µg/mL had lower cytotoxicity compared to commercial vectors for the delivery of non-viral genes. The O-GNRs–plasmid-DNA complexes were absorbed by the vesicular structures of dividing HeLa and HUVEC cells, released into the cytoplasm of the cell and entered the nucleus. In these cells, the O-GNRs–plasmid-DNA complexes increased the efficiency of gene delivery and transfection.

Dong et al. [72] proposed a GNR-based nanocarrier grafted by polyethyleneimine (PEI-g-GNR) as an efficient gene vector. The nanoribbons were synthesized by the longitudinal unpacking of MWCNTs treated subsequently with strong acids and ultrasound in order to obtain surface carboxylic acid groups for PEI grafting by electrostatic assembly. It was assumed that PEI-g-GNR protected locked nucleic acid modified by molecular beacon (LNA-m-MB) probes from nuclease digestion or interaction with a single-stranded binding protein. The cytotoxicity and apoptosis caused by PEI-g-GNR were negligible under optimal transfection conditions. In combination with the high specificity of LNA to microRNA, the developed delivery system was proposed for transferring modified LNA into cells for target RNA recognition.

The study [73] is devoted to the use of O-GNRs coated by PEG-1,2-distearoyl-sn-glycero-3-phosphoethanolamine-N-[amino(polyethylene glycol)] (DSPE) (O-GNR–PEG-DSPE) as an agent for the delivery of antitumor drugs into glioblastoma multiformae cells (U251). The antitumor drug, being an APE-1 endonuclease inhibitor, was applied to the O-GNR–PEG-DSPE complex using a simple non-covalent method. The results showed that the absorption of O-GNR–PEG-DSPE by U251 cells exceeded 67% and the increase in APE-1 expression in cells after 24 h was 60%. The MCF-7 (human breast cancer cell line)- and CG-4 (rat glial progenitor cells)-type cells absorbed 38% and 29% of the used oxidized GNRs, respectively. The TEM analysis of U251 showed large aggregates of O-GNR–PEG-DSPE in the vesicles. The O-GNR–PEG-DSPE functionalized by the antitumor drug was highly toxic for U251, but had low or no toxicity for MCF-7 and CG-4 cells [74].

Ligands, such as peptides, antibodies, or other epitopes, bind and activate specific cellular receptors. These substances are used for the targeted cellular delivery of drugs, genes, and imaging agents. Several studies [75,76] are devoted to the activation of epidermal growth factor receptors (EGFRs) by O-GNRs non-covalently functionalized with PEG-DSPE. This activation generates a predominantly dynamin-dependent macropinocytosis-like response and results in the significant absorption of O-GNR–PEG-DSPE by cells with a high EGFR expression. The authors also reported that cells with an integrated human papillomavirus (HPV) genome show the increased internalization of O-GNR–PEG-DSPE due to the modulation of the EFGR-activated viral protein E5 [68].

Sphingolipids [77,78] can be used as possible anticancer agents [79,80,81] due to their powerful proapoptotic effects. However, due to their extreme hydrophobic properties, there is currently no clinically approved in vivo delivery method for these therapeutic agents. Suhrland et al. [82] developed a new method for loading ceramide C6 onto O-GNRs and graphene nanoplates (GNPs). The mass spectrometry analysis showed loading efficiencies of 57% and 51.5%, respectively. The cytotoxicity analysis showed that at 100 μg/mL ceramide-loaded O-GNRs and GNPs, HeLa cell viability was reduced by 93% and 76% compared to the untreated HeLa cells. It should be noted that nanoparticles at the same concentration without C6 ceramide did not significantly affect cell viability. The authors found that the cytotoxicity had an apoptotic nature. The confocal images of live cells with fluorescent NBD–ceramide ((6-((N-(7-nitrobenz-2-oxa-1,3-diazol-4-yl)amino)hexanoyl)sphingosine)) loaded onto the O-GNRs showed sustained uptake over 30 min.

Thus, GNRs are of interest as gene and drug delivery vectors (Table 2) and possibly also in tissue engineering and bioimaging [33]. However, the high hydrophobicity and toxicity of GNRs require further efforts to increase their biocompatibility in case of in vivo applications. 

## 3. Biocompatibility and Toxicity

### 3.1. Biocompatibility

Currently, there are several works in which the cytological effects of GNRs have been investigated. At the same time, very few authors have studied the biocompatibility of GNRs. For example, Liu et al. [84] conducted experiments on the cultivation of human medulloblastoma cells (DAOY) with nitrogen-doped GNRs aerogels (N-GNRA) obtained by self-assembly in combination with thermal annealing. Amino groups were grafted to the surface of GNRs using an epoxy ring-opening reaction. A high level of nitrogen doping (7.6 atm.%) was achieved during heat treatment as a result of functionalization and GNRs edge effects. Three-dimensional N-GNRAs had a hierarchical porous structure. The quasi-one-dimensional GNRs acted as the building blocks for making GNRs mesh sheets, which additionally created three-dimensional wireframes with micrometer-scale pores. The GNRs edge effect combined with nitrogen doping and porosity resulted in good electrical conductivity and superhydrophilicity. The study showed the good biocompatibility of the N-GNRA that opens up its opportunities for biomedicine applications. 

MCF-7 exposed to PEG-DSPE-coated O-GNRs (O-GNR–PEG-DSPE) and 80 μM lucanthone-loaded O-GNR–PEG-DSPE do not exhibit decreased viability compared to untreated controls after 24 h exposition [73]. In [85], the authors showed the absence of the toxic effect of O-GNRs noncovalently functionalized with DSPE to the components of the blood vascular system at concentrations of 20 μg/mL. In addition, one-hour exposure to O-GNR–PEG-DSPE did not induce histamine release from mast cells, PF4 activation in platelets, and complement activation. However, at every studied concentration, there was a slight decrease (5–10%) in the levels of anti-inflammatory cytokines. 

The analysis of the cytotoxicity of films consisting of chitosan, alginate, and 2.5 wt % GNRs (CHI/f–GNR–ALG) obtained by unzipping MWCNTs on the mouse fibroblasts (L929) showed the sample cytocompatibility. In particular, L929 is able to proliferate on the surface of CHI/f–GNR–ALG (200 µL) [86]. On this basis, the authors suggested that biopolymer films containing GNRs have potential applications for wound healing, as well as the engineering of the heart and bone tissue.

Foreman et al. [87] analyzed the viability of adherent HEK 293T epithelial cells and non-adherent A20 cells incubated with O-GNRs. According to the study, there was no change in cell viability after the 24 h or 48 h incubation of the cells at O-GNRs concentrations up to 100 mg/mL. 

The authors of reference [88] investigated the biocompatibility of O-GNRs modified with phospholipid-PEG (PL–PEG) in vivo. Doxorubicin-loaded GNRs has 6.7-fold lower IC_50_ values for chemo-photothermal therapy toward U87 glioma cells than the IC_50_ values in traditional chemotherapy. They found that PL–PEG–O-GNRs was excreted from the body via the renal pathway in the urine, and the hematological analysis showed that this nanomaterial was not toxic. The authors consider PL-PEG-O-GNRs as potential nanocarriers of drugs to develop an effective cancer treatment strategy that will not only increase the effectiveness of therapy, but also reduce the risk of side effects of the nanocarrier in the body.

Thus, there is evidence that the cytotoxicity of GNRs can be significantly reduced by their edge or surface chemical modification. For example, doping with nitrogen, oxidation, coating with biocompatible polymers such as PEG or chitosan give an obvious increase in biocompatibility. The hydrophilization of carbon nanomaterials is a versatile strategy to provide their bioavailability and applicability in biomedicine. This is especially important to take into account when developing bioengineering constructs based on GNR, which should have minimal cytotoxicity.

### 3.2. Toxicity

According to Mbeh et al. [89], O-GNRs synthesized using the oxidative unpacking of MWCNTs and functionalized with albumin at a concentration of 100 μg/mL have high cytotoxicity towards human epithelial cells, causing the inhibition of proliferation and the induction of apoptosis.

The results of the study [90] showed that O-GNRs with PEG-DSPE have heterogeneous cell-specific cytotoxicity. The screening of the O-GNR–PEG-DSPE cytotoxicity was performed using cervical cancer cells (HeLa), mouse fibroblasts (NIH-3T3), as well as breast cancer cells SKBR3 and MCF7. In general, all the cells showed a dose-dependent (10–400 μg/mL) and time (12–48 h) decrease in viability. However, the toxicity for MCF7 or SKBR3 cells was significantly lower than that for HeLa cells. MCF7 and SKBR3 remained 100% viable for up to 48 h for at the O-GNR–PEG-DSPE concentration of 10 μg/mL, and the viability level dropped to ~78% at the maximum concentration of 400 μg/mL. For HeLa, a significant cell death (5–25%) was observed already at a low concentration of 10 μg/mL, and was also observed with an increase in concentration (CD50 ≥ 100 μg/mL). The toxicity to HeLa has been associated with a higher uptake of O-GNR–PEG-DSPE compared to those to other cell types.

The same authors showed that O-GNR–PEG-DSPE at concentrations of 20, 80, and 160 μg/mL caused a low concentration-dependent deformation of erythrocytes, which did not lead to hemolysis [85]. There was also a significant uptake of O-GNR–PEG-DSPE by endothelial cells and a concentration-dependent decrease in their viability. The cytotoxicity of O-GNRs obtained from single-walled CNTs was analyzed on human neuroblastoma cell lines SK-N-BE(2) and SH-SY5Y by assessing cellular reactive oxidative stress, mitochondrial membrane potential, expression of lysosomal proteins, and cell growth [91]. The results showed that O-GNRs at low concentrations increase reactive oxygen species (ROS) production and induce autophagy in both cell lines within hours of exposure; however, these effects are not accompanied by growth arrest or cell death.

In the study [92], the cytotoxicity of O-GNRs (310 × 5000 nm) and oxidized graphene oxide nanoparticles (O-GNPs; 100 × 100 nm) obtained by oxidative treatment of MWCNTs (100 × 5000 nm) and stacked graphene nanofibers (SGNFs; 100 × 5000 nm) were studied. The evaluation in vitro showed a higher cytotoxicity of O-GNRs compared to that of O-GNPs. The authors suggested that the effect is associated with a large number of carbonyl groups, as well as an increased O-GNRs length, i.e., the strong toxic effect of O-GNRs is the result of a synergistic effect between these two factors. In addition, the carbon source used to prepare oxidized graphene must be considered in biological research.

Chowdhury et al. [93] demonstrated that the ultrasonic bath or probe induces GNR structural disruption on MCF7 and A549 cells (human adenocarcinoma alveolar basal epithelial cells). According to the results, a GNR suspension of 20 μg/L treated with an ultrasonic probe for 1 min could cause a significant decrease in the overall metabolic state of cells compared to bath-treated or untreated suspension. The structural analysis showed that the ultrasonic probe treatment results in the disruption of the GNR structure and the formation of fine carbon “debris”, which may be the cause of toxicity.

A comparative analysis of the toxicity of single-layer reduced O-GNRs (rO-GNRs) and reduced graphene oxide sheets (rGOS) in relation to human mesenchymal stem cells (hMSCs) showed significant cytotoxic effects of rO-GNR at a concentration of 10 μg/mL of after 1 h of exposure, while rOGS showed the same degree of toxicity at a concentration of 100 μg/mL after 96 h. The main mechanism of rGOS action was assumed to be oxidative stress, which causes minor damage to the cell membrane. While the outflow of RNA from hMSCs showed that neither the formation of reactive oxygen nor significant damage of the membranes cells can explain the destruction of cells caused by rO-GNRs. The results also showed that rO-GNRs could penetrate cells and cause DNA fragmentation and chromosomal aberrations even at a low concentration of 1.0 μg/mL, after a short exposure time of 1 h [94,95].

Talukdar et al. [96] investigated the effect of graphene nanostructures with various morphologies, such as O-GNRs, graphene oxide nanoplatelets (O-GNPs), and graphene nano-onions (GNOs)) on the toxicity and differentiation potential of hMSCs. The cells were treated with various concentrations (5–300 μg/mL) of nanomaterials for 24 and 72 h. The results showed the dose-dependent, time-independent cytotoxicity of graphene nanostructures at concentrations above 50 μg/mL. The cellular uptake of GNOs and O-GNPs was shown by TEM and confocal Raman spectroscopy; no such effects were observed for O-GNRs.

For the imaging and phototherapy of human glioblastoma (U87MG), Akhavan et al. functionalized rO-GNRs with polyethylene glycol (rO-GNR–PEG). Cytotoxic and genotoxic effects on the cells depended on the concentration of rO-GNR–PEG. When the cells were incubated in the dark with 100 μg/mL of rO-GNR–PEG for 24 h, more than 72% of cell death and more than 29% of DNA fragmentation were observed. At a lower concentration (1 μg/mL), cell death and DNA fragmentation decreased to about 11% and 7%, respectively [97,98].

According to the considered researches on cyto- and genotoxicity, the possible mechanisms of the toxic action of GNRs can be suggested mechanical damage to cell membranes; ROS production, which leads to the inhibition of proliferation, induction of apoptosis, autophagy, and DNA fragmentation; chromosomal aberrations (Table 3).

It should be noted that the studies of the toxicity of GNRs for the whole organism have not yet been carried out, excluding the work of Lu et al. [88]. However, it is known that other carbon nanomaterials have various toxic effects on living organisms, including neurotoxicity, nephrotoxicity, hepatotoxicity, genotoxicity and epigenetic toxicity, and dermatotoxicity [99,100,101,102,103]. This indicates the importance of conducting similar studies on GNRs.

## 4. GNRs in the Environment

Similar to other carbon nanomaterials, GNRs can enter the environment not only as part of the nanoindustry products, but also during natural processes, for example, during the combustion of organic matter. There is no information on the environmental toxicity of GNRs for ecosystems in the analyzed researches. An exception is the work of Lalwani et al., which investigates the oxidative biodegradation of O-GNRs and rO-GNRs by lignin peroxidase (LiP) [104]. LiP is an enzyme secreted by white rot fungi (Phanerochaete chrysosporium), which are widespread throughout the world in forest soils with dead and decaying organic matter. LiP breaks down lignin contained in the plant cell wall. The TEM and Raman spectroscopic analysis of O-GNRs and rO-GNRs treated with LiP for 4–96 h showed holes formation, confirming the structural degradation of graphene sheets. O-GNRs showed a higher rate of biodegradation compared to rO-GNRs: within 4 h after processing, numerous holes with a diameter of 1–5 nm were found on the O-GNRs sheets, which increased to ~300–350 nm after 48 h. The hole diameter on the rO-GNRs sample was 5–30 nm after 48 h of enzymatic treatment. After 96 h, the O-GNRs appeared to have completely degraded, while numerous holes were observed to extend across the entire width of the rO-GNR sample. These results showed that rO-GNRs can undergo oxidative biodegradation by LiP under environmental conditions [105].

The studies that are concerned with the closest chemical and structural analogues of GNRs–CNTs and graphene indicate their significant effects on various types of living organisms. A large number of studies demonstrate the toxic effects of carbon nanomaterials in relation to bacteria [106,107,108], fungi [109,110], protozoa [111], algae [112,113,114], higher plants [115,116], roundworms [117,118,119], arthropods [120], and mammals [121,122,123,124]. In addition, the possibility of bioaccumulation of carbon nanomaterials in living organisms has been investigated [125,126,127,128].

Based on this, it can be assumed that GNRs can also have a significant impact on components of ecosystems, especially microorganisms, and it can be bioaccumulated and migrate within ecosystems. These issues certainly warrant further study.

## 5. Conclusions

GNRs demonstrate the greatest prospects in the field of biomedicine, particularly in creating nanodevices for the biomolecules detection and single-molecular techniques. In addition, GNRs are of interest as gene and drug delivery vehicles. There are researches that prove the possibility of using GNRs in tissue engineering.

Practical applications of GNRs in direct contact with the human body are limited by their potential toxicity. A number of researchers have noted the damaging effects of GNRs, including cytotoxicity and genotoxicity. Moreover, the toxicity of nanoribbons is higher than those of such chemical analogs as O-GNPs. According to several studies, the possible mechanisms of toxicity are as follows: the ability to induce ROS production and autophagy; inhibition of proliferation; induction of apoptosis; DNA fragmentation; chromosomal aberrations. However, other authors have not confirmed any toxic effects of GNRs in experiments with human cell lines. The discrepancy in the observed results can be explained by differences in the methods of synthesis and structure of GNRs, their functionalization by different groups, and the use of different concentrations. In addition, different cell lines exhibit individual responses to exposure to nanomaterials. Furthermore, in order to obtain consistent results, the unification of the nanotoxicological experimental conditions is needed.

The chemical modification of the surface of GNRs with hydrophilic groups can significantly increase their bioavailability and biocompatibility.

There is an evidence of the biodegradability of GNRs in the environment. By analogy with other carbon nanomaterials, GNRs can be toxic to living organisms of various species. They can be bioaccumulated and migrate through ecosystems, but there are currently no data on this.

In general, this review allows concluding that GNRs, as components of high-precision nanodevices and therapeutic agents, have good prospects for application in biomedicine, but the limiting factors for the use of GNRs are their high hydrophobicity and insufficiently studied toxicity. There is a lack of information on the effect of GNRs on the whole organism using in vivo experiments, as well as on environmental toxicity, accumulation, migration, and destruction within ecosystems.

## Figures and Tables

**Table 1:** GNRs in biomedical devices.

Functionalized Material	Devices	Detection Method	Detection Limits	References
GNRs with nanopores	DNA sequenser	Electrochemical	-	[51]
GNRs with nanopores	DNA sequenser	Electrochemical	-	[54]
GNRs with nanopores	DNA sequenser	Electrochemical	-	[55,56]
Metallic GNRs with zigzag edges (ZGNRs) with a nanopore in its inner part	DNA sequenser	Electrochemical	-	[57]
Cytosine-functionalized GNRs	DNA sequenser	Electrochemical	-	[58]
GNRs decorated with iron oxide (Fe_3_O_4_) nanoparticles	DNA sensor	Electrochemical	-	[59]
GNRs with gold nanocages (AuNCs@GNRs)	DNA sensor	Electrochemical	1 fM–100 pM	[60]
Template enzyme glucose oxidase with GNRs	Electrochemical biosensor of glucose	Electrochemical	20 mg/L	[61]
Hybrid GNR/Ag NPs	Plasmonic sensing platform for the sequential colorimetric detection of dopamine and glutathione	Colorimetric	0.46 μM for dopamine and 1.2 μM for glutathione	[62]
Chevron-like GNRs	Electrochemical sensor of epinephrine	Differential pulse voltammetry	2.1 × 10^−6^ M	[63]

**Table 2:** Delivery of genes and drugs.

Functionalized Material	Component for Delivery	Cells	Effect	References
O-GNRs (20–60 μg/mL)	Enhanced green fluorescence protein plasmid or siRNA against glyceraldehyde-3-phosphate dehydrogenase (GAPDH)	HeLa and HUVEC	Concentration- and time-dependent increase in gene delivery and gene transfection efficiencies up to 96–98%	[71]
GNR-based nanocarrier grafted by polyethyleneimine	Locked nucleic acid modified by molecular beacon (LNA-m-MB)	HeLa	The efficient transfer of LNA-m-MB into cells for the recognition of the target miRNA has been demonstrated.	[72]
O-GNRs coated by PEG-DSPE	Antitumor drug Lucanthone	U251	Uptake by U251 cells exceeding 67% and 60% in APE-1-overexpressing U251 post 24 h	[73]
O-GNRs coated by PEG-DSPE	Doxorubicin-	HeLa	Epidermal growth factor receptors (EGFRs) are activated and are taken up in significant amounts in cells with high EGFR expression.	[75]
O-GNRs (100 µg/mL)	C6 ceramide	HeLa	Decrease in cell viability by 93%. O-GNRs without C6 ceramide did not significantly reduce cell viability.	[82]
O-GNRs coated by PEG-DSPE (5–40 μg/mL)	C16 and C24 ceramides	HeLa	Significant biological effects in cells in conjunction with C6 ceramide and UV irradiation treatment. O-GNRs themselves have a number of significant biological effects that interfere with the ability of long-chain ceramides to sensitize or protect cells from pro-apoptotic stressors.	[83]

**Table 3:** Toxicity of GNRs according to various studies.

Material	Physical-Chemical Properties and Functionalization	Object	Dose and Exposure Time	Effect	References
GNRs	Nitrogen-doped GNR aerogels	Human medulloblastoma (DAOY)	-	Biocompatible sample	[84]
GNRs	Multilayer films consisting of chitosan, alginate and 2.5 wt % GNRs	Mouse fibroblasts (L929)	1, 3, and 7 days	Cytocompatible sample	[86]
O-GNRs	-	Adhesive epithelial cells (HEK293T) and non-adherent cells (A20)	Up to 100 mg/mL; 24 and 48 h	No effect	[87]
O-GNRs	Functionalized with albumin	Human epithelial cells	100 μg/mL	High cytotoxicity. Inhibition of proliferation and induction of apoptosis	[89]
O-GNRs	Functionalized with PEG-1,2-distearoyl-sn-glycero-3-phosphoethanolamine-N-[amino (polyethylene glycol)] (DSPE)	Cervical cancer cells (HeLa), mouse fibroblasts (NIH-3T3), and breast cancer cells (MCF7)	10–400 μg/mL; 12–48 h	Dose-, time-, and cell-dependent effects. MCF7 or SKBR3 were 100% viable up to 48 h at 10 μg/mL and reduced viability to 78% at 400 μg/mL. For HeLa cells, a 5–25% decrease in viability was observed even at a low concentration of 10 μg/mL.	[90]
O-GNRs	Functionalized with PEG-DSPE	Erythrocytes, endothelial cells	20, 80, and 160 μg/mL	The concentration-dependent deformation of erythrocytes did not lead to hemolysis. The uptake of nanomaterials by endothelial cells and a concentration-dependent decrease in their viability	[85]
O-GNRs	-	Human neuroblastoma SK-N-BE (2) and SH-SY5Y		Increased reactive oxidative stress (ROS) production and the induction of autophagy within hours of exposure	[91]
rO-GNRs	-	Human mesenchymal stem cells (hMSCs)	1 and 10 μg/mL; 1 h	Significant cytotoxic effects. rO-GNRs can enter cells and cause DNA fragmentation and chromosomal aberrations even at low concentrations	[94,95]
rO-GNRs	Functionalized with polyethylene glycol (r O-GNR–PEG)	Human glioblastoma (U87MG)	100 μg/mL; 24 h	More than 72% of cell death and more than 29% of DNA fragmentation	[97,98]
